# Testicular Traction Technique with Intact Cremasteric Reflex, a Novel Approach for Manual Detorsion: Case Report

**DOI:** 10.5811/cpcem.1568

**Published:** 2024-05-29

**Authors:** Garrett Trang, Taz Brinkerhoff

**Affiliations:** The University of Arizona, College of Medicine-Phoenix, Phoenix, Arizona

**Keywords:** *case report*, *testicular torsion*, *manual detorsion*, *technique*, *cremasteric reflex*

## Abstract

**Introduction:**

Recognizing testicular torsion is extremely important in patients presenting to the emergency department (ED) with acute scrotal pain. Traditional manual detorsion techniques are frequently employed by emergency physicians but are not always successful. Delays in detorsion increase the risk of testicular infarction and infertility, and the need for orchiectomy. Novel techniques such as the testicular traction technique have been described as a potential solution for difficult-to-detorse testicles.

**Case Report:**

Our case report describes a 20-year-old male with no significant past medical history who presented to a rural ED with acute, atraumatic testicular pain secondary to testicular torsion with an intact cremasteric reflex. After confirming the diagnosis using Doppler ultrasound, manual detorsion using the traditional “open book” technique was attempted and unsuccessful. The patient was subsequently successfully detorsed using the novel testicular traction technique.

**Conclusion:**

The testicular traction technique is a safe, rapid, and effective primary or adjunctive technique in manual testicular detorsion. Given the time-sensitive nature of testicular torsions, adjunctive techniques play a crucial role in managing challenging detorsions, particularly in resource-limited rural settings with limited access to urologic services. Although it is commonly thought that the cremasteric reflex is absent in testicular torsions, it may be present in rare circumstances, and its presence should not be an absolute in ruling out torsion.

Population Health Research Capsule What do we already know about this clinical entity?

*The testicular traction technique was first described in 2022 as an adjunctive manual detorsion technique for testicular torsions.*

What makes this presentation of disease reportable?

*A patient with a testicular torsion and an intact cremasteric reflex was detorsed using the testicular traction technique.*

What is the major learning point?

*This is a safe, noninvasive treatment that provides rapid pain relief. Absence of the cremasteric reflex may not always be a reliable sign of testicular torsion.*

How might this improve emergency medicine practice?

*The testicular traction technique is an effective primary or adjunctive technique for difficult-to-detorse testicles.*

## INTRODUCTION

Scrotal complaints comprise at least 0.5% of all emergency department (ED) visits.^1^ Among the differential diagnoses, testicular torsion is one of the most serious and is considered a surgical emergency. The annual incidence of testicular torsion is estimated to be 3.8 per 100,000 for males <18 years of age, and rates of orchiectomy were found to be as high as 42% in these boys.^2^ ^,^ ^3^ Given that the complications of testicular torsion include testicular infarction and infertility, quickly diagnosing and treating it is imperative.^3^ ^,^ ^4^

In the ED, manual detorsion is a safe, rapid, noninvasive treatment that should be attempted to reverse ischemia and provide rapid pain relief.^4^ Since up to 95% of testicular torsions are due to internal rotation, the detorsion technique often requires external rotation of the testicle as if one was “opening a book.”^5^ For a suspected torsion of the left testicle, the physician should place his or her right thumb and index finger on the testicle and rotate 180 degrees from medial to lateral (or clockwise).^4^ The procedure may be repeated several times since torsion can involve rotations of 180–720 degrees.^4^ The success rate of manual detorsion in the literature varies; it can be as high as 76–95% when performed by urologists, but other studies found the success rate more variable.^6^ ^–^ ^8^ Given the variable success rate of the “open book” technique, it is important to have an adjunctive technique if it fails or urologic intervention is not immediately available.

The testicular traction technique was first described in 2022 by Mellick et al^9^; it involves grasping the testicle with one or both hands and pulling inferiorly to stretch the spermatic cord to its maximum length. From there, manual detorsion of the testicle is performed by externally rotating the testicle using the “open book” technique until the spermatic cord feels normal. We describe a successful detorsion using the testicular traction technique after conventional methods failed.

## CASE REPORT

A 20-year-old White male with no known medical conditions presented to a rural ED with complaints of atraumatic and continuous pain to the left testicle and inguinal region that worsened with movement. The pain developed after a day of work involving frequent heavy lifting. During his shower at home approximately five hours prior to presentation, he noticed the left testicular pain was more pronounced with associated bulging and tenderness in the left scrotal region. He denied any associated symptoms such as nausea and vomiting. He had no history of torsion, undescended testicles, testicular malignancy, or surgery to the testicle or abdominal region.

**Image 1.**
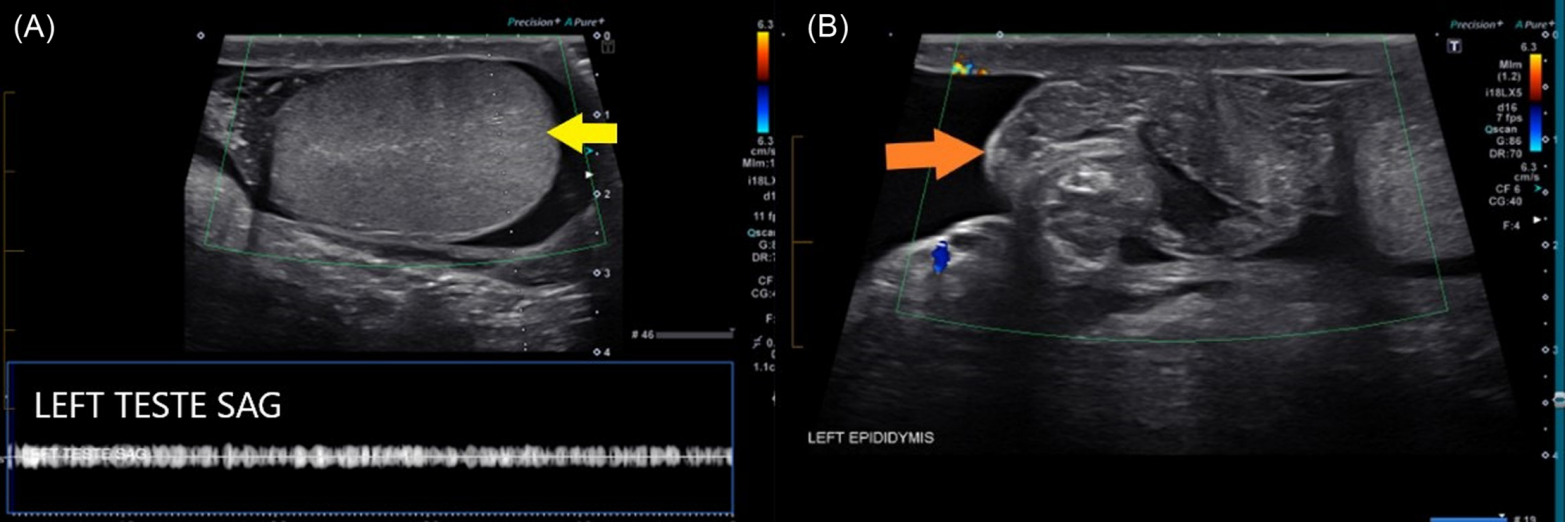
Arterial and venous flow in the left testicle and epididymis under Doppler ultrasound. Note the lack of flow in both the left testicle (yellow arrow) (A) and left epididymis (orange arrow) (B). There was a heterogenous mass (orange arrow) superior to the left testis without blood flow, consistent with tangled vasculature (B). A discussion with the radiologist determined that the spectral waveform was artifact and not venous flow (A). *TRANS*, transverse view; *SAG*, sagittal view.

On physical exam, the patient appeared pale, nervous, and apprehensive of movement. Vital signs were within normal limits. Genitourinary examination revealed a high-riding and hard testicle that was diffusely tender to palpation. There were no masses in the inguinal region, and no testicular erythema, swelling, visible masses, or penile discharge. A hard, knot-like mass was palpated on the superior pole of the left testicle along the spermatic cord, suspicious for a torsed spermatic cord. Unexpectedly, the cremasteric reflex was present. The sonographer who performed the ultrasound approximately six hours after symptom onset noted minimal blood flow to the left testicle (Image 1). The on-call radiologist quickly confirmed the diagnosis of testicular torsion. The radiology report also noted a tangled, heterogenous mass superior to the testicle that was consistent with the torsed spermatic cord palpable on physical exam (Image 1). There was no notable enlargement or changes in echotexture that were concerning for an infarction or necrosis.

The patient was prepped for manual detorsion in a standing position approximately six hours after the onset of symptoms. We attempted the “open book” technique several times in both directions without successful detorsion. Subsequently, the testicular traction technique (Figure) was performed by first grasping and applying inferior traction to the left testicle, while correcting the side-lying angle to a more natural vertical angle of the testicle.

**Image 2.**
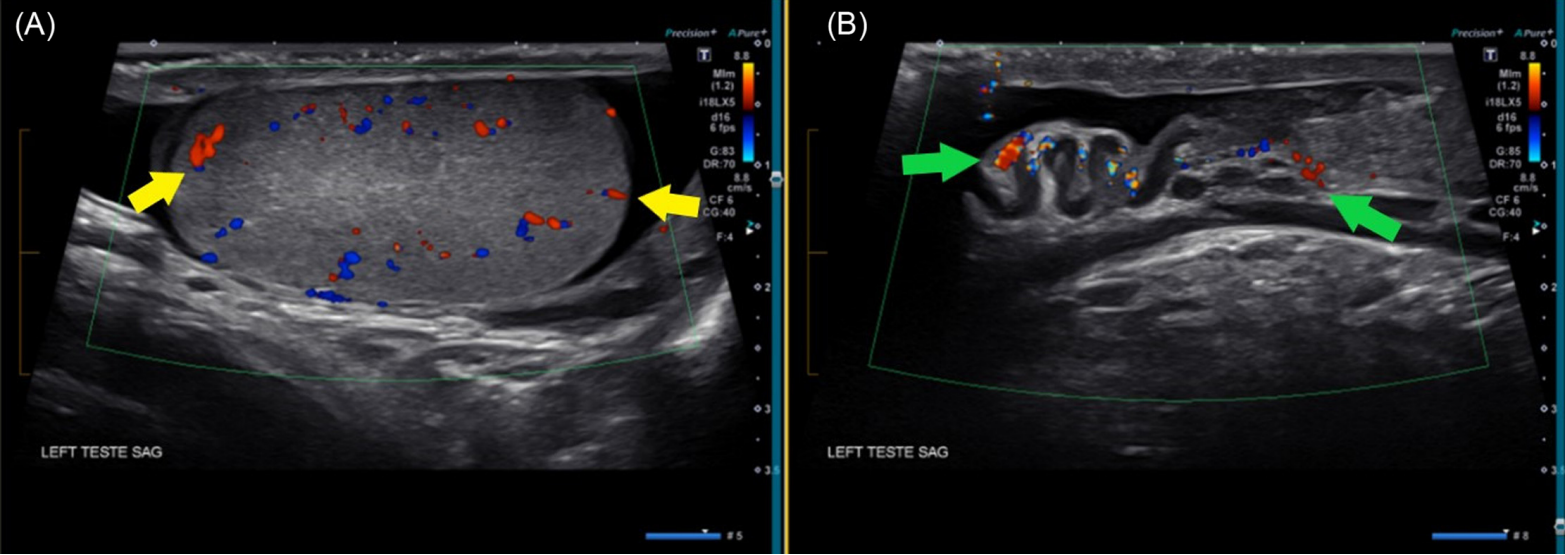
Repeat Doppler ultrasound of the left testicle after manual detorsion. (A) Sagittal view of the left testicle showing restored arterial and venous flow (yellow arrows). (B) Sagittal view of the spermatic cord and epididymis with resolution of the heterogenous mass and restoration of blood flow (green arrows), consistent with successful detorsion.

**Figure.**
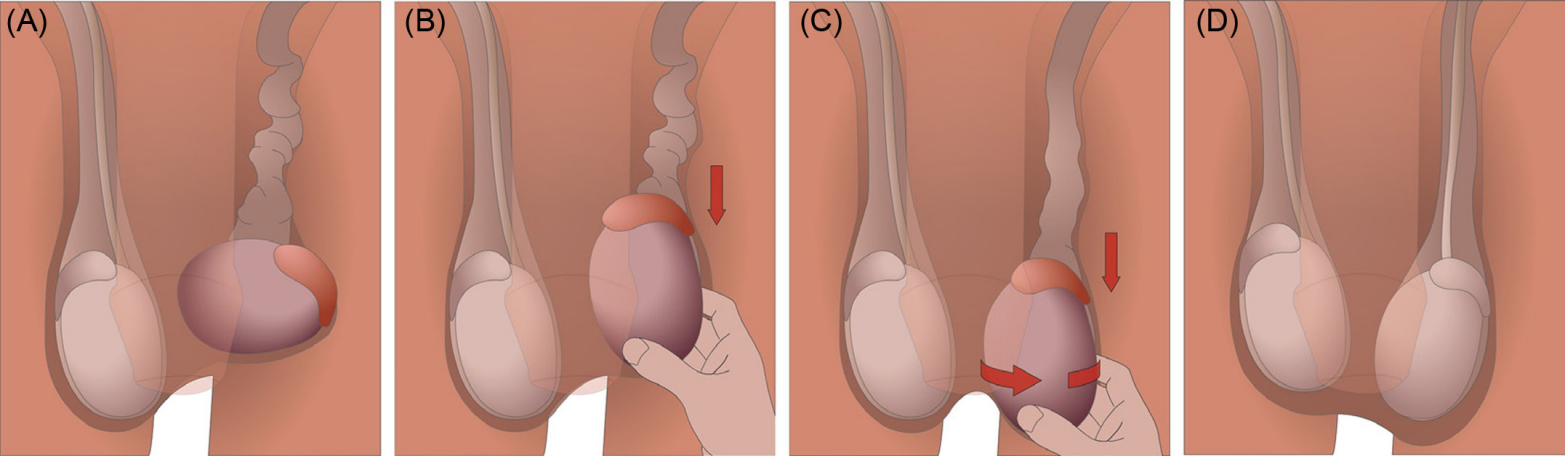
The testicular traction technique. (A) The torsed left testicle is identified. Typical symptoms include a high-riding, hard, inflamed, and tender testicle as depicted here. A twisted spermatic cord may also be palpated superior to the testicle. (B) Traction is applied to the affected testicle until the spermatic cord is maximally lengthened. In our case, the side-lying angle of the torsed testicle was corrected to a more natural, vertical angle. (C) With the spermatic cord lengthened, the testicle is rotated externally while maintaining traction. Most testicular torsions are medially twisted; therefore, clockwise rotation should be attempted for the left testicle and counterclockwise rotation for the right testicle. The physician should visualize and palpate for any flipping or spinning of the testicle throughout the maneuver. (D) Successful detorsion is often represented by immediate resolution of pain. Repeat Doppler ultrasound should be performed to confirm restoration of blood flow to the affected testicle.

The tension reduced the palpable lump of anticipated spermatic cord at the superior pole. While maintaining traction and lengthening the spermatic cord, the left testicle was manually rotated clockwise by ∼90°. At this point, the testicle spontaneously detorsed by at least 1–2 turns. It was challenging to determine the precise number of rotations, as the testicle rapidly rotated within the scrotum. However, the physician could feel the testicular anatomy during this movement. Resolution was apparent by both visible and physical exams immediately following the procedure. The patient reported instant relief of pain. A repeat Doppler ultrasound was performed, which confirmed the detorsion and restoration of normal blood flow with no evidence of infarct or necrosis (Image 2). Based on resolution of symptoms and blood flow, the on-call urologist determined that the patient could follow up in an outpatient setting urgently for elective orchiopexy procedure. Follow-up information was not available.

## DISCUSSION

This case reports describes a 20-year-old man with a testicular torsion and intact cremasteric reflex that was successfully detorsed with the testicular traction technique. The spermatic cord is highly mobile and stretches and retracts under normal physiologic responses. As a result, the traction maneuver in the testicular traction technique should be physiologically and anatomically tolerated.^9^ This technique is particularly useful when torsion has been prolonged and the spermatic cord becomes edematous, increased in volume, and partially trapped making extraction from the inguinal canal necessary.^9^ The proposed mechanism of the technique is analogous to stretching a tangled phone cord to allow for partial unraveling prior to applying a rotational force. Scheier and Levy in 2023 described a similar phenomenon where each traction attempt returned transient and pulsatile blood flow to the testicle despite the manual detorsion failing.^10^

Our case was consistent with what was described in the first case series reporting use of this technique.^9^ The traction technique in our case was employed as an adjunct maneuver, which removed the resistance felt during the initial detorsion attempt using standard methods. While the stretching of the spermatic cord did not directly lead to unraveling of the torsion, spontaneous unraveling did occur after gentle external rotation of the testicle. We hypothesize that the additional downward force of gravity in the standing position may have contributed to the spontaneous detorsion. However, the technique could be performed in a supine position as described in the previous case series.

The most important determinants of an early salvage rate of testes are the time from symptom onset to detorsion and the degree of spermatic cord twisting.^11^ The sensitive time frame of testicular salvage in testicular torsion cases further emphasizes the importance of timely diagnoses and manual detorsion. In rural hospitals like ours, subspeciality services such as urology are not immediately available, and transfer to tertiary care centers may prolong testicular ischemia if not successfully detorsed in the ED. The cremasteric reflex is often thought to be absent in 100% of patients with testicular torsion. Unexpectedly, the cremaster reflex was present in our case, which is similar to what was reported in other case reports.^12^ The cremasteric reflex may be a useful sign, but its reliability in diagnosing or excluding testicular torsion has been challenged in recent years.^13^

Regardless, a scrotal ultrasound should be immediately performed on any patient who presents with acute atraumatic scrotal pain, and manual detorsion should be attempted in a timely fashion. The rates of orchiectomy following testicular torsion vary in the literature; nonetheless, they are significantly higher in patients who were not successfully manually detorsed.^14^ Manual detorsion in the ED can buy precious time that is vital for successful surgical salvage and may sometimes even convert an emergent urologic surgery into an urgent but elective one.^6^

The time window for survival and successful salvage of a torsed testicle had been commonly thought to be 6–8 hours or less.^15^ Delays past this time window may lead to orchiectomy, which is associated with reduced fertility and medico-legal litigation. Some physicians even forgo manual detorsion beyond this point. In a systematic review by Mellick et al that included 2,116 patients with testicular torsion, they found that testicular survival was significantly beyond the original 6–8 hour time frame: 90.4% survival when detorsed within the first 12 hours, 54% from 13–24 hours, and 18.1% when after 24 hours. Successful detorsion may still be achieved past the commonly accepted window, but the decision to attempt manual detorsion should be made on a case-by-case basis.

## CONCLUSION

The absence of the cremasteric reflex may not always be a telltale sign in identifying patients with an acute scrotum, and ultrasound should be performed whenever testicular torsion is suspected. It is commonly thought that testicles are unsalvageable past the 6–8 hour time window; however, there is literature that supports longer survivability. Physicians should not forgo manual detorsion solely based on the duration of torsion. The testicular traction technique is a rapid, easy-to-perform, and safe maneuver that can be used as a primary or adjunctive treatment for detorsion in any setting. Despite limited patient cases, the novel testicular technique shows promise as a solution for difficult-to-reduce testicles.
